# A Protocadherin-Cadherin-FLRT3 Complex Controls Cell Adhesion and Morphogenesis

**DOI:** 10.1371/journal.pone.0008411

**Published:** 2009-12-22

**Authors:** Xuejun Chen, Eunjin Koh, Michael Yoder, Barry M. Gumbiner

**Affiliations:** Department of Cell Biology, University of Virginia Health Sciences Center, Charlottesville, Virginia, United States of America; University of Birmingham, United Kingdom

## Abstract

**Background:**

Paraxial protocadherin (PAPC) and fibronectin leucine-rich domain transmembrane protein-3 (FLRT3) are induced by TGFβ signaling in *Xenopus* embryos and both regulate morphogenesis by inhibiting C-cadherin mediated cell adhesion.

**Principal Findings:**

We have investigated the functional and physical relationships between PAPC, FLRT3, and C-cadherin. Although neither PAPC nor FLRT3 are required for each other to regulate C-cadherin adhesion, they do interact functionally and physically, and they form a complex with cadherins. By itself PAPC reduces cell adhesion physiologically to induce cell sorting, while FLRT3 disrupts adhesion excessively to cause cell dissociation. However, when expressed together PAPC limits the cell dissociating and tissue disrupting activity of FLRT3 to make it effective in physiological cell sorting. PAPC counteracts FLRT3 function by inhibiting the recruitment of the GTPase RND1 to the FLRT3 cytoplasmic domain.

**Conclusions/Significance:**

PAPC and FLRT3 form a functional complex with cadherins and PAPC functions as a molecular “governor” to maintain FLRT3 activity at the optimal level for physiological regulation of C-cadherin adhesion, cell sorting, and morphogenesis.

## Introduction

PAPC is a downstream target of TGF-beta (activin/nodal) signaling that is required to mediate activin-induced down-regulation of C-cadherin mediated cell adhesion and tissue morphogenesis in gastrulating *Xenopus* embryos [1]. Recently, FLRT3 and its downstream effecter RND1 were also found to be induced by activin and required for down-regulation of C-cadherin mediated cell adhesion and tissue morphogenesis in *Xenopus* [2]. Interestingly, PAPC, FLRT3 and RND1 share very similar expression profiles in developing *Xenopus* embryos, all being highly expressed at the involuting mesoderm that undergoes dramatic morphogenetic cell movements during gastrulation [2]–[4]. These similarities suggest that PAPC and FLRT3 may work cooperatively in regulating cell adhesion and tissue morphogenesis. Therefore we have examined the functional and physical relationships between PAPC and FLRT3 as well as their interactions with C-cadherin. The structures of PAPC and FLRT3 as well as mutant constructs used in this study are shown in Figure S1.

## Results and Discussion

### FLRT3 Inhibits C-Cadherin Adhesion Activity but Mediates Cell Sorting Only when Expressed at Low Levels

**Figure 1:**
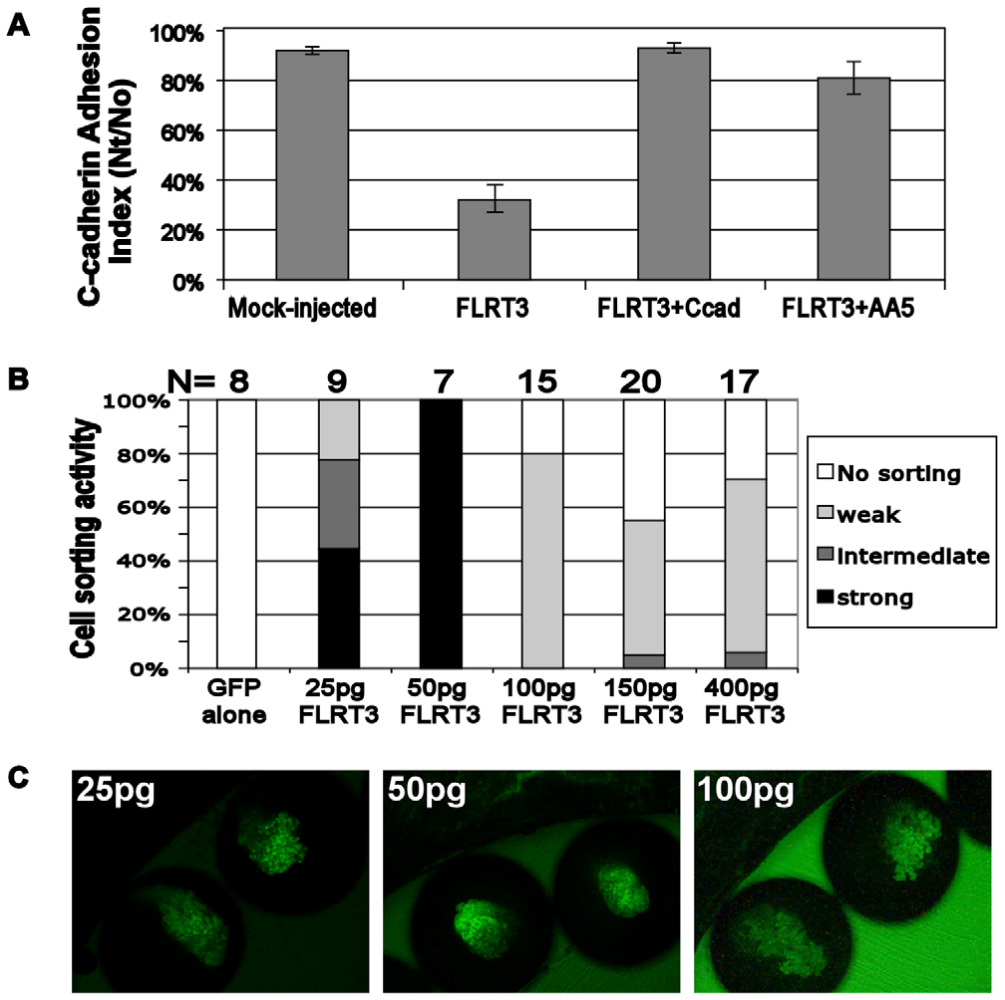
FLRT3 inhibits C-cadherin adhesion activity and induces cell sorting at low expression levels. A) The effect of FLRT3 expression on blastomere adhesion to C-cadherin coated substrates. Blastomeres were collected from stage 9 embryos that were mock-injected (as control), injected with FLRT3 RNA (160 pg) alone, or co-injected with FLRT3 RNA (160 pg) and C-cadherin RNA (1.5 ng). A portion of the FLRT3 expressing blastomeres was further treated with the Fab fragment of C-cadherin activating antibody, AA5 (1 µg/ml), for 30 min. Nt/No  =  the ratio of the number of blastomeres remaining attached to the C-cadherin substrate after shaking to the number before shaking. B) Dose effects of FLRT3 on its cell sorting activity. Different amounts of FLRT3 RNA, together with NLS-GFP mRNA (200 pg) for a lineage tracer, were injected into one animal blastomere of embryos at the 16-cell stage. The cell sorting activity of FLRT3 was evaluated at stage 13 by observing how much the GFP labeled cells disperse into the uninjected region. C) Representative cell sorting images of embryos injected with 25 pg, 50 pg or 100 pg of FLRT3 RNA.

We first tested whether FLRT3 specifically inhibits C-cadherin mediated cell adhesion in a manner similar to PAPC. FLRT3-expressing blastomeres showed significantly lower adhesion to purified C-cadherin coated substrates (Figure 1A), consistent with previous results using E-cadherin as adhesion substrate [2]. This inhibition by FLRT3 is specific because it can be reverted either by overexpression of C-cadherin or by treatment with the specific C-cadherin activating antibody, AA5 (Figure 1A), similar to the regulation of C-cadherin by PAPC [1]. We have shown previously that both activin and PAPC regulate C-cadherin adhesion activity without altering its protein levels at the cell surface [1], [5]. In contrast, Ogata et al. reported that FLRT3, which is also downstream of activin, inhibited C-cadherin mediated adhesion by stimulating the internalization of C-cadherin into the cell [2]. However, in our experiments employing both trypsin sensitivity assays (Figure S2A) and surface biotinylation assays (Figure S2B and S2C), FLRT3 overexpression did not significantly affect C-cadherin levels at the cell surface, similar to activin and PAPC. Moreover, immunofluorescence staining of C-cadherin in the involuting mesoderm, where both FLRT3 and PAPC are expressed endogenously, showed no decrease in C-cadherin staining at cell-cell contacts compared to the ectodermal or endodermal regions (Figure S2D). The extensive internalization of C-cadherin observed by Ogata et al. [2] might be a secondary event due to a more severe or prolonged loss of cadherin mediated adhesion caused by prolonged and higher activin or FLRT3 expression, since disengaged cadherin molecules are known to be more susceptible to endocytosis [6]–[8]. Even Ogata et al. acknowledged that their activin treatment, injecting activin RNA into embryos at the 2-cell stage, has a much stronger and prolonged effect than treating isolated blastula-stage blastomeres with a controlled low concentration (5 ng/ml) of activin for 1 hr [2].

**Figure 2:**
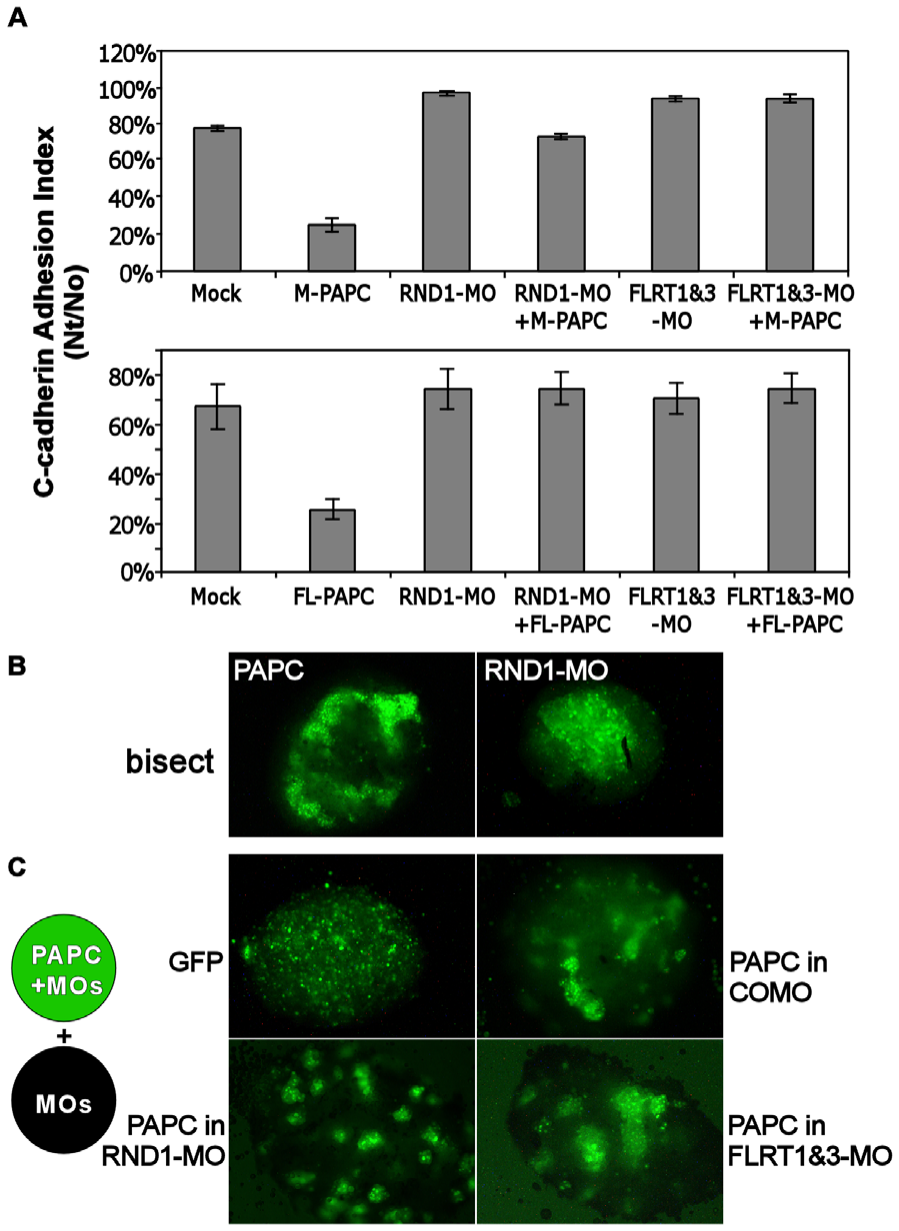
Effects of FLRT and RND1 knockdowns on PAPC mediated cell adhesion regulation and cell sorting. A) FLRT morpholinos (FLRT1&3-MOs) and RND1 morpholino (RND1-MO) increase C-cadherin mediated cell adhesion and counteract PAPC-mediated down-regulation of C-cadherin adhesion activity. Embryos were injected as indicated (FL-PAPC or M-PAPC RNA: 1.5 ng, FLRT or RND1 morpholinos: 80 ng) at the 2-4 cell stage. Blastomeres were collected at stage 9 for adhesion assays on C-cadherin coated substrates. B) Comparison of PAPC induced cell sorting versus RND1-MO induced cell sorting by cell reaggregation assay. Either FL-PAPC RNA or RND1-MO was co-injected with NLS-GFP RNA into 2–4 cell stage embryos. At stage 9, dissociated animal cap blastomeres from injected (labeled) embryos were coaggregated with blastomeres from mock injected embryos. The aggregates were bisected and examined under a fluorescence microscope. C) PAPC induces cell sorting in the absence of FLRTs or RND1. FLRT1&3-MOs (80 ng) or RND1-MO (80 ng) were injected into all embryos at the 2–4 cell stage to make all cells deficient in FLRT1&3 or RND1. FL-PAPC RNA (1.5 ng/embryo) and NLS-GFP RNA (or NLS-GFP alone as a control) were injected into one half of the embryos to determine if labeled cells would sort from unlabeled cells. Cell reaggregation assays were performed between labeled (green) and unlabeled cells.

**Figure 3:**
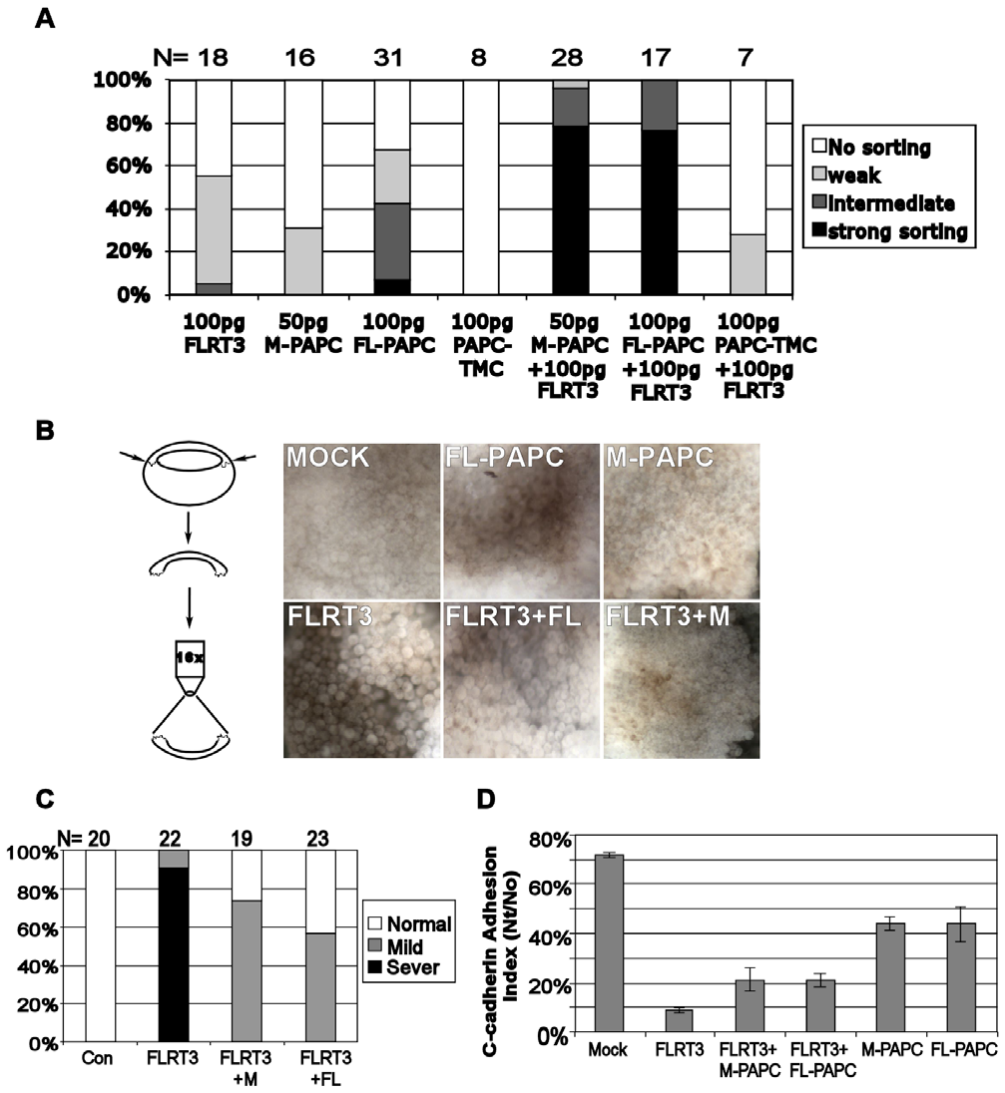
Effects of PAPC expression on the cell sorting and adhesion regulating function of FLRT3. A) PAPC and FLRT3 cooperate in causing cell sorting. Cell sorting (cell dispersal) assays were performed by injecting 100 pg of FLRT3 RNA, 50 pg of M-PAPC RNA, 100 pg of PAPC-TMC or 100 pg of FL-PAPC RNA separately or in combination into one animal blastomere at the 16 cell stage along with NLS-GFP RNA. B) PAPC expression suppresses the FLRT3-induced disintegration of the blastocoel roof. The indicated RNAs (FLRT3 RNA: 0.4 ng, FL-PAPC or M-PAPC RNA: 1.6 ng) were injected into the animal hemispheres of the 2-4 cell stage embryos, the animal cap explants were excised at stage 9, and the cells of the blastocoel roof were examined and photographed as shown in the diagram. C) Graphical summary of 3 independent experiments examining the PAPC-rescue of the FLRT3 phenotype as shown in B. The results were categorized in terms of severe, mild or no cell adhesion disruption. D) PAPC expression suppresses the downregulation of C-cadherin mediated cell adhesion by FLRT3. Blastomere adhesion assay on C-cadherin coated substrates were performed with cells from embryos injected as in B.

Since PAPC mediates cell sorting by down-regulating C-cadherin adhesion activity, we asked whether FLRT3 also mediates cell sorting. Overexpression of FLRT3 (200–400 pg RNA/embryo) severely disrupted cell adhesion, causing blastomeres to round up and dissociate from each other [2]. These FLRT3 expressing cells exhibited very little cell sorting activity (Figure 1B and 1C, at ≥100 pg), presumably due to the disruption of the tissue integrity that is necessary for cells to rearrange within the tissue. This is in contrast to PAPC overexpression (1.5 ng RNA/embryo), which exhibits strong cell sorting activity [1] (Figure 2B and 2C) but never causes cells to round up and dissociate or compromise tissue integrity [1] (Figure 3B) despite its reproducibly measurable inhibition of C-cadherin mediated adhesion [1] (Figure 2A and 3D). Importantly, low levels of FLRT3 expression, insufficient to cause gross tissue disruption, did cause strong cell sorting behavior (Figure 1B and 1C). Therefore, FLRT3 appears to be a more potent C-cadherin inhibitor than PAPC, and at low expression levels that modulate adhesion without disrupting tissue integrity FLRT3 mediates cell sorting, similar to PAPC.

### FLRT3 and PAPC Functionally Interact in the Regulation of Cell Adhesion and Cell Sorting

Because PAPC and FLRT3 similarly down-regulate C-cadherin cell adhesion, we asked whether the function of either of them is dependent on the other. Exogenous FLRT3 expression disrupts cell adhesion at stage 9 [2] (Figure 1 and 3) even before PAPC starts to be expressed [1], [3]. Along with the observation that PAPC morpholinos did not block FLRT3 function (data not shown), these findings show that FLRT3 functions independent of PAPC.

To determine whether PAPC function requires FLRT3 we need to consider all three FLRT proteins, FLRT1, FLRT2 and FLRT3, which are expressed in *Xenopus* embryos and can all disrupt cell adhesion by recruiting RND1 [2]. FLRT1 is maternally expressed and its expression persists in the animal hemisphere until gastrulation [2]. FLRT3 is mainly expressed in the dorsal marginal zone and involuting mesoderm during gastrulation [2]. FLRT2 is expressed later at neurula stages [2] and therefore does not need to be considered at the stage that our experiments are performed. Therefore, we eliminated the function of FLRT1 and FLRT3 proteins by either knocking down their expression with morpholinos or by knocking down the expression of RND1, the downstream effector of FLRT proteins, as done previously [2]. Although the effectiveness and specificity of these morpholinos had been described before [2], we still tested the efficacy of FLRT3 morpholinos (FLRT3-MO) since we had anti-FLRT3 antibody. FLRT3-MO knocked down exogenously expressed FLRT3 by over 90% in Xenopus embryos (Figure S3). In addition, FLRT1 morpholinos and RND1 morpholinos all caused gastrulation defects (data not shown) as described previously [2], suggesting that they were effective. Co-injection of FLRT1&3 morpholinos (FLRT-MO) or RND1 morpholinos (RND1-MO) with PAPC-RNA, counteracted the down-regulation of C-cadherin mediated adhesion that is observed with PAPC expression alone (Figure 2A). A cytoplasmic-domain-deleted PAPC-mutant (M-PAPC, see Figure S1) can down-regulate cadherin-based cell adhesion [1], [9] and induce cell sorting [1], [3], [9] as well as full-length PAPC (FL-PAPC). RND1-MO and FLRT-MO had the same effects on M-PAPC induced cell adhesion decrease and cell sorting as on FL-PAPC (Figure 2A), suggesting that the absence or presence of the cytoplasmic tail of PAPC does not affect the relationship of PAPC with FLRT3 and RND1.

However, it has been reported that knocking down FLRT or RND1 expression with morpholinos increases cell adhesion in the embryo [2]. Similarly, we find that blastomeres from FLRT-MO or RND1-MO injected embryos adhered better to C-cadherin substrates compared to mock-injected control embryos (Figure 2A), and cells from RND1-MO injected embryos sort to the center of cell aggregates when mixed with uninjected control cells (Figure 2B, right panel). In contrast, PAPC expressing cells sort to the periphery of aggregates [1] (Figure 2B, left panel). Cells with higher adhesion are known to sort to the inside of aggregates with cells having lower adhesion [10]–[13]. Therefore, RND1 or FLRT knockdown may counteract the affects of PAPC expression simply because they affect adhesion in the opposite direction of PAPC, rather than being required downstream of PAPC.

To unambiguously determine whether FLRT and RND1 are required for PAPC function, we examined whether PAPC can still induce cell sorting among cells that are uniformly deficient in RND1- or FLRT- expression. RND1 or FLRT expression was knocked down with morpholinos in all the cells used for the cell reaggregation sorting assays, and PAPC was expressed only in one half of the cells (also labeled with GFP expression). Despite the absence of RND1 or FLRTs, PAPC still caused strong cell sorting (Figure 2C). Because the cell sorting activity of PAPC depends on its ability to decrease C-cadherin adhesion [1], [9], these results demonstrate FLRT1, FLRT3 and RND1 are not required for PAPC to mediate adhesion regulation, and that PAPC and FLRT3 probably function in parallel to dynamically regulate cadherin-mediated cell adhesion during gastrulation.

Despite the finding that they appear to be able to function independent of each other, PAPC and FLRT-RND1 may nonetheless function coordinately in the regulation of cell adhesion and sorting. We therefore examined how co-expression of PAPC and FLRT3 affect each other's cell sorting activity. As described previously (Figure 1B and 1C), high levels of FLRT3 expression did not cause strong cell sorting (Figure 3A, 100 pg FLRT3) because they disrupted tissue integrity that is necessary for cell sorting (Figure 3B). Also, low levels of PAPC expression, which do not significantly inhibit C-cadherin-mediated adhesion, did not cause cell sorting (Figure 3A, 50 pg M-PAPC); intermediate levels of PAPC caused only modest sorting (Figure 3A, 100 pg FL-PAPC). However, when high amounts of FLRT3 RNA were co-injected with the low or intermediate amounts of PAPC RNA, the cells exhibited very strong cell sorting activity compared to embryos expressing either protein alone (Figure 3A). As control, PAPC-TMC, an extracellular domain deleted PAPC mutant that is inactive in regulating C-cadherin mediated adhesion or inducing cell sorting [1], [9] (also see Figure 3A, 100 pg PAPC-TMC), did not show the cooperation with FLRT3 in inducing cell sorting like FL-PAPC and M-PAPC (Figure 3A). Although they can regulate adhesion independently, the cooperative effects of FLRT3 and PAPC in cell sorting suggest that they might function together *in vivo* to optimally regulate cell adhesion, cell sorting and morphogenetic cell movements during gastrulation.

We then investigated the reason for this cooperation between PAPC and FLRT3. A clue came from the observation that FLRT3 only mediates cell sorting activity when expressed at low levels that do not cause cell dissociation (Figure 1B and 1C). We therefore asked whether PAPC could modulate the adhesion disrupting activity of FLRT3. Injection of 400 pg FLRT3 RNA into early embryos lead to severe disruption of cell adhesion at the blastula stage and causes blastomeres to completely dissociate from the blastocoel roof (Figure 3B), as observed previously [2]. When animal cap explants were excised, most of the inner layer cells were dissociated and rounded up (Figure 3B). However, when either M-PAPC or FL-PAPC was co-expressed with FLRT3, the cell dissociation phenotype was significantly rescued, and was limited to only a very small area immediate around the injection sites (Figure 3B). The quantitative results from several independent experiments summarized in Figure 3C demonstrate that PAPC reproducibly rescues the FLRT3 induced cell dissociation phenotype, irrespective of the presence or absence of its cytoplasmic tail. The extracellular domain-deleted FLRT3 mutant, FLRT3-TMC (also known as FLRT3-ΔLRR, see Figure S1), also disrupts cell adhesion similar to FLRT3 [2]. As expected, FL-PAPC and M-PAPC also rescue the FLRT3-TMC induced cell dissociation phenotype (Figure S4B-S4C and S4E-S4F). In contrast, PAPC-TMC failed to rescue the phenotype (Figure S4D and S4H). This result not only provides a good specificity control for the rescue by FL-PAPC and M-PAPC in addition to the GFP control (Figure S4A and S4D), but also suggests that the extracellular domain of PAPC is required for its ability to counter FLRT3 function.

We confirmed that the suppression of FLRT3-induced cell dissociation by PAPC is due to its effects on C-cadherin-mediated adhesion. Although both PAPC and FLRT3 inhibit C-cadherin adhesion activity, with FLRT3 causing more severe inhibition, co-expression of PAPC reduced the magnitude of the effect of FLRT3 on C-cadherin mediated cell adhesion (Figure 3D). This moderating effect of PAPC on FLRT3 function therefore produces a more physiological change in adhesion that allows cells to remain integrated in the tissue and to rearrange and sort out within the confines of the tissue. We propose that PAPC functions as a molecular “governor” to limit the activity of the more potent cell adhesion inhibitor, FLRT3, and together they decrease cadherin-mediated cell adhesion in an optimal range for morphogenesis.

### PAPC, FLRT3 and Cadherin Physically Interact

The similar expression profiles and the functional interactions between PAPC and FLRT3 suggest that they may physically interact. Moreover, since both proteins regulate C-cadherin adhesion activity, they may physically interact with cadherins. Although we were able to detect interactions between C-cadherin and PAPC and FLRT3 in embryo lysates using chemical crosslinking (Figure S5), the signal was very weak (although reproducible) due to interference with the method by the large amounts of yolk proteins. Therefore, we decided to investigate the potential interactions between PAPC, FLRT3 and a classical cadherin in tissue culture cells using both bead recruitment assay and co-immunoprecipitation (co-IP) assay.

**Figure 4:**
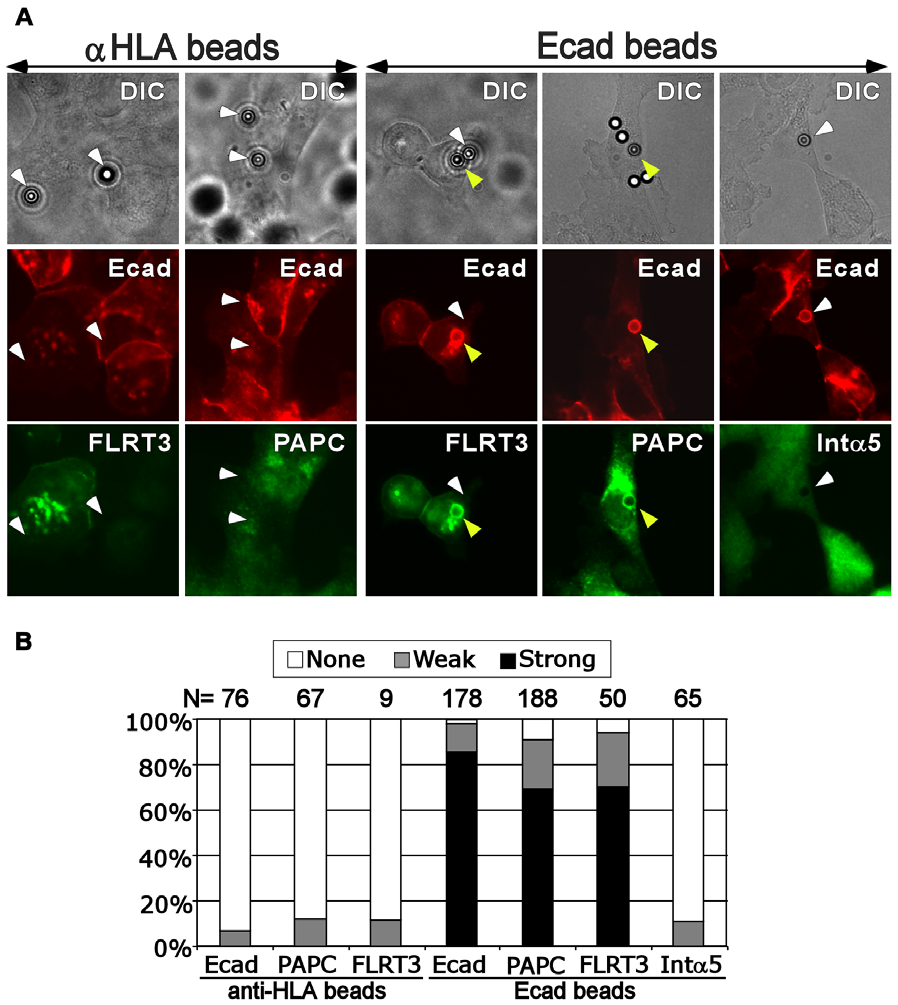
PAPC, FLRT3 and E-cadherin physically interact shown by bead recruitment assay. A) E-cadherin coated beads specifically recruit PAPC and FLRT3, but not integrin α5 (Intα5), at the surface of transfected A431 cells. Protein A-coupled microbeads were coated with purified human E-cad-EC·Fc and attached to sub-confluent A431 cells that express *Xenopus* PAPC and/or FLRT3. Double immunofluorescence staining against E-cadherin (red) and PAPC (green), E-cadherin (red) and FLRT3 (green), or E-cadherin (red) and integrin α5 (green). Anti-HLA coated protein A beads were used as negative control. The positions of beads determined by DIC microscopy are marked with arrowheads. White arrowheads indicate beads that did not recruit at least one of the stained proteins, whereas the yellow ones point to those that did recruit E-cadherin, PAPC, and/or FLRT3. B) Quantification of the recruitment of E-cadherin, PAPC and FLRT3 by both anti-HLA control beads and E-cadherin beads as well as the recruitment of integrin α5 by E-cadherin beads.

**Figure 5:**
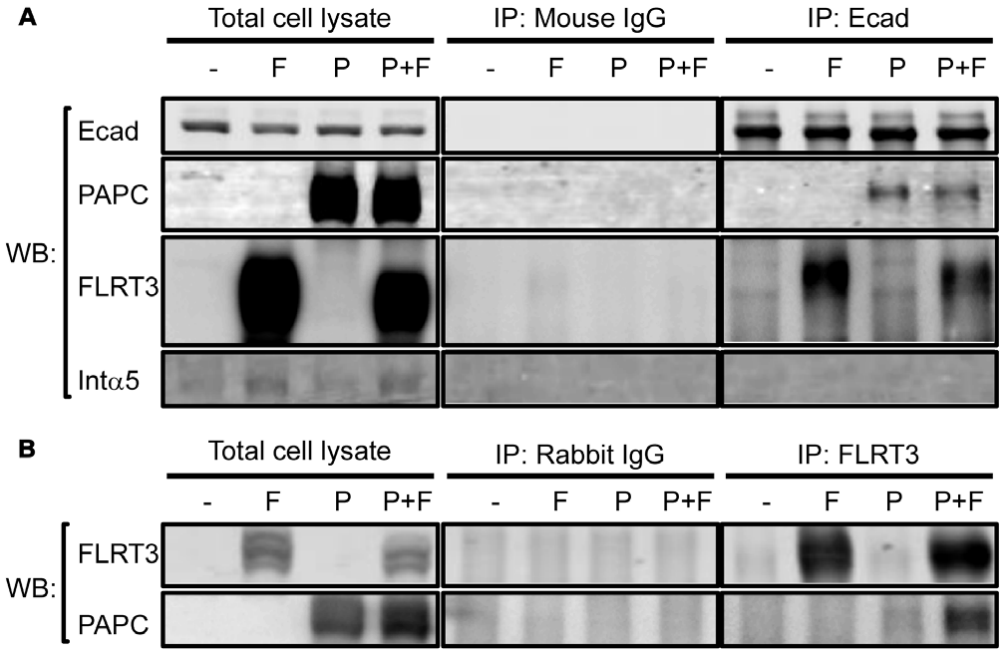
PAPC, FLRT3 and E-cadherin physically interact shown by co-immunoprecipitation (co-IP) assays. A) PAPC and FLRT3 co-IP with E-cadherin in transfected A431 cells. Parental A431 cells and A431 cells exogenously expressing FLRT3 alone, PAPC alone, or both were grown to confluence in 10 cm petri dishes, lysed in lysis buffer, and IPed with mouse IgG or anti-E-cadherin IgG and analyzed by Western blots against E-cadherin, PAPC, FLRT3 or integrin α5 (as negative control). B) PAPC co-IPs with FLRT3. The same cells as in A) were lysed in lysis buffer, and IPed with rabbit IgG or anti-FLRT3 IgG and analyzed by Western blots for FLRT3 and PAPC.

Co-recruitment of cell surface proteins to ligand-coated beads has been employed widely as an assay to detect interactions between membrane proteins [14]–[16]. For these experiments, we used A431 tissue culture cells transfected to express PAPC (stable) and FLRT3 (transient). Because they express endogenous human E-cadherin, microbeads coated with purified human E-cadherin-Fc protein [14], [15] were used for the ligands. It is reasonable to expect that these interactions may occur with different classical cadherins, because the rat PAPC homolog arcadlin has been found to similarly regulate N-cadherin in neurons [17], and we have been able to detect an interaction between PAPC and E-cadherin by co-IP from detergent extracts of A431 cells stably expressing PAPC (Figure 5A). Microbeads coated with either human E-cadherin or with anti-HLA antibody (as a cell binding negative control) were attached to A431 cells expressing PAPC, FLRT3 or both, and cells were washed, fixed and subject to double immunofluorescence staining: red fluorescence for E-cadherin cytoplasmic domain (not present in the ligand) and green fluorescence for either PAPC or FLRT3 (Figure 4A). The E-cadherin (Ecad) beads not only efficiently recruited endogenous E-cadherin to the beads as expected, but also efficiently recruited PAPC and FLRT3 (Figure 4A: the 3^rd^ and 4^th^ columns and Figure 4B: the 5^th^ and 6^th^ columns). In contrast, the control anti-HLA beads bound to the cells but did not recruit E-cadherin, PAPC, or FLRT3 (Figure 4A: the left two columns and Figure 4B: the left three columns). As another control, integrin α5 was not recruited by E-cadherin beads (Figure 4A: the 5^th^ column and Figure 4B: the 7^th^ column; Figure S6 demonstrates the effectiveness of the integrin α5 antibody in immunofluorescence). Therefore both PAPC and FLRT3 can specifically associate with E-cadherin at the surface of A431 cells.

We then examined whether PAPC, FLRT3 and E-cadherin directly interacts with one another by co-IP experiments. We transiently transfected either parental A431 cells or A431 cells that stably express Xenopus PAPC with Xenopus FLRT3 plasmids. We then IPed either E-cadherin (Figure 5A) or FLRT3 (Figure 5B) from parental A431 cells (-) and A431 cells that express FLRT3 alone (F), PAPC alone (P) or both (P+F). IPs with mouse IgG (Figure 5A) and rabbit IgG (Figure 5B) were used as controls. Significantly, both PAPC and FLRT3 co-IP with E-cadherin in A431 cells, irrespective of the presence of the other protein (Figure 5A: F, P and F+P). Neither protein was detected in the mouse IgG control IPs; moreover, endogenously expressed integrin α5 did not co-IP with E-cadherin. Therefore, both PAPC and FLRT3 can each specifically interact with E-cadherin independent of the other. Nonetheless, a significant amount of PAPC co-IPed with FLRT3 in A431 cells expressing both proteins (Figure 5B), suggesting that PAPC also interacts somehow with FLRT3, either directly or mediated by E-cadherin. In either case, our data indicate that PAPC, FLRT3 and E-cadherin co-exist in the same protein complex. It is very likely, therefore, that PAPC, FLRT3 and C-cadherin also form a complex in *Xenopus* embryos that explain the functional interactions between these proteins.

### PAPC Inhibits FLRT3 Activity by Reducing Its Recruitment of RND1

**Figure 6:**
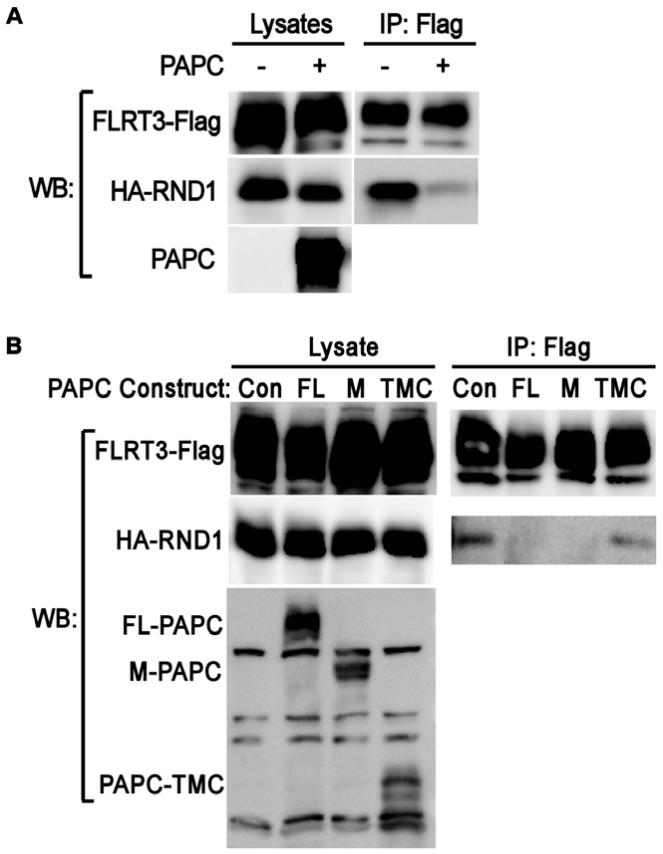
PAPC expression inhibits the binding of RND1 to FLRT3. A) Effect of full length PAPC on the binding of RND1 to FLRT3. FLRT3-Flag RNA (0.3 ng) and RND1-HA RNA (0.15 ng) was co-injected into 4-cell stage embryos with or without FL-PAPC RNA (1.5 ng) and extracts of embryos were subject to anti-Flag IP and Western blotting (WB) for the proteins shown. B) The extracellular and transmembrane domains of PAPC inhibit FLRT3 recruitment of RND1. Expression of FL-PAPC and M-PAPC, but not PAPC-TMC, in the *Xenopus* embryo inhibits FLRT3 recruitment of RND1. Co-IP and Western blot analysis were done as in A.

We investigated the mechanism by which PAPC governs FLRT3 activity. FLRT3 functions by recruiting a downstream effector RND1, a constitutively active small GTPase, to its cytoplasmic domain [2]. Therefore we asked whether PAPC expression affects the ability of FLRT3 to recruit RND1 (Figure 6). Flag-tagged FLRT3 (FLRT3-Flag) was co-expressed with HA-tagged RND1 (HA-RND1) in *Xenopus* embryos with or without the co-expression of PAPC. PAPC expression significantly reduced the amount of HA-RND1 co-IPed with FLRT3-Flag (Figure 6A). Because recruitment of RND1 is necessary for FLRT3-mediated inhibition of cell adhesion, PAPC probably inhibits FLRT3 activity by limiting its ability to recruit RND1.

Different regions of PAPC and FLRT3 seem to be required for their C-cadherin adhesion regulating activity. The transmembrane domain plus cytoplasmic domain of FLRT3 (FLRT3-TMC) has been shown to disrupt cell adhesion in *Xenopus* embryos as well as full-length FLRT3 [2], presumably due to its ability to recruit RND1. For PAPC, the extracellular domain plus transmembrane domain are required for its regulation of C-cadherin [1], [9]. FLRT3-TMC caused cell dissociation as previously observed [2], and FL-PAPC, M-PAPC, but not PAPC-TMC, rescued FLRT3-TMC induced cell dissociation (Figure S4). Thus, PAPC can regulate the activity of FLRT3 that is missing its extracellular domain, but the extracellular domain of PAPC is required. Consistent with the functional interactions, M-PAPC and FL-PAPC, but not PAPC-TMC, inhibited the recruitment of RND1 by FLRT3, as assayed by co-IP experiments (Figure 6B). Therefore, the domains of PAPC involved in limiting FLRT3 activity are the same as those required for its regulation of C-cadherin.

We have identified a new mechanism by which PAPC regulates the cell sorting and morphogenetic cell movements during gastrulation in *Xenopus* embryos. PAPC, FLRT3 and RND1 are all expressed at the dorsal marginal zone and involuting mesoderm in response to the Nodal signaling during gastrulation, and they are likely to form a functional protein complex with the more broadly expressed C-cadherin protein. FLRT3 recruits the RND1 GTPase to the cell surface, where it antagonizes RhoA, disrupts cortical actin cytoskeleton network, and thereby strongly interferes with C-cadherin mediated cell-cell adhesion [2], [4], [18], [19]. While PAPC also directly down-regulates cadherin mediated adhesion at more moderate levels, it also acts as a molecular “governor” or “buffer” to restrain the activity of FLRT3 through the inhibition of the recruitment of RND1. This ensures that the decrease in the cell adhesion is optimal for cell rearrangements without being destructive to tissue integrity.

We do not know the exact molecular details by which PAPC governs FLRT3 activity, but based on our knowledge of the protein domains involved and the biochemical properties of the two proteins, we offer the following model. FLRT3 is known to form homodimers, probably mediated by the transmembrane domain [20] and we speculate that only dimerized FLRT3 can recruit RND1 and disrupt cell adhesion. PAPC also forms oligomers (probably trimers), but these depend on the extracellular domain [9]. We speculate that only oligomeric forms of PAPC transmembrane domains are competent for its interactions with FLRT3 transmembrane domain. If oligomers of PAPC inhibit dimerization of FLRT3 via interactions between their transmembrane domains, recruitment of RND1 by FLRT3 would be blocked, leading to an inhibition of FLRT3 signaling. This hypothesis explains why M-PAPC and FL-PAPC, but not PAPC-TMC, inhibit the function of FLRT3 and FLRT3-TMC.

The existence of the PAPC-FLRT3-cadherin complex and the mechanisms of adhesion regulation uncovered in this study may be general and applicable to other tissues. PAPC, FLRT3 and RND1 are all expressed in other tissues that all express various classical cadherins, including neurons, and are implicated in neuronal morphology and activity [17], [21], [22], and it will be important to examine their functional interactions in the regulation of cell adhesion in these other tissues.

## Materials and Methods

### *Xenopus* Embryo Handling, Cell Adhesion, Cell Sorting Assays

The preparation, handling, and staging of *Xenopus laevis* embryos and the microinjection of mRNAs or morpholinos into *Xenopus* embryos have been described [1], [23], [24]. The blastomere adhesion assay on C-cadherin substrates and the cell sorting assays, including cell reaggregation assay and cell dispersal assay, were done as previously described [1], [9]. The blastomeres used for the adhesion and reaggregation assays were the non-pigmented inner layer ectodermal cells dissociated from the animal cap explants excised from injected or uninjected stage 9 embryos.

### Constructs, Morpholinos and Antibodies

Plasmids for making synthetic FL-PAPC, M-PAPC and PAPC-TMC RNAs and anti-PAPC mAb 28F12 have been described [1], [3], [9]. For expression of flag-tagged PAPC, complementing flag-tag oligos were annealed and cloned into the C-terminal end of PAPC coding sequence in pCS2+ vector. Plasmids pCS2+/FLRT3, pCS2+/FLRT3-flag, pCS2+/FLRT3-TMC (FLRT3-ΔLRR) and pCS2+/HA-RND1, as well as morpholinos to FLRT1 and FLRT3, and polyclonal anti-FLRT3 antibody were gifts from Dr. Ken Cho at University of California, Irvine and have been described [2]. Morpholinos to PAPC and RND1 were synthesized by Gene Tools, Inc as described [1], [2], [25]. Anti-Flag M2 mAb was obtained from Sigma. A scheme of the constructs used in this study is shown in Figure S1.

### Biochemical Analyses of Cadherin and FLRT3

Trypsinization and biotinylation of surface C-cadherin were performed as previously described [1], [9]. For co-immunoprecipitation (co-IP), A 431 and FL-PAPC stably transfected A431 cells were transfected with pCS2+/FLRT3 utilizing Lipofectamine 2000 according to the manufacturer's protocol. After 24 hrs, cells were lysed with lysis buffer (50 mM Tris-HCl, pH 7.4, 150 mM NaCl, 1% NP40 with Roche protease inhibitor cocktail) and centrifuged at 15,000×g for 10 minutes. Rabbit IgG, mouse IgG, FLRT antibody or E-cadherin antibody (BD bioscience) was added to the supernatants that had been precleared with protein A or protein G agarose. Cell lysate and antibody mixtures were incubated at 4°C with end-to-end rotation overnight, then with protein A or protein G agarose for two more hours. The beads were washed four times with lysis buffer, resuspended with 2x SDS sample buffer, and boiled to elute proteins. Anti –FLRT3, anti-E-cadherin, anti-PAPC, and anti-integrin alpha5 (Abchem) were utilized for Western blot analysis.

### Bead Recruitment Assay

Human E-cadherin extracellular domain fused with human IgG Fc (hE-Fc) was purified from conditioned media of CHO cells stably expressing hE-Fc as previously described [14], [26], [27]. The bead recruitment assay was done as described previously [14] with minor modification. Protein A-coated 5.5 µm beads (Bangs Laboratories, Inc., Fisher, IN) were incubated with hE-Fc or anti-HLA antibodies in binding buffer (25 mM Boric acid, 150 mM NaCl, 1 mM CaCl_2_, 0.5 mM MgCl_2_, and 0.001% Tween 20, pH 8.0) for two hours at 4°C, and subsequently blocked with human Fc fragment in 1% BSA in PBS++ (PBS supplemented with 1 mM CaCl_2_, 0.5 mM MgCl_2_) for 1 hr at 4°C. The bead suspension was sonicated for 5 secs and allowed to attach to cells for 2 hrs. Cells were fixed with 4% paraformaldehyde and permeabilized with ice-cold methanol. Anti-E-cadherin (BD Biosciences, San Jose, CA), anti-FLRT, and anti-PAPC antibodies were used for immunofluorescence staining.

## Supporting Information

Figure S1 Scheme of different protein constructs used in this study. FL-PAPC: the full-length PAPC contains a signal peptide, 6 cadherin ectodomains, a transmembrane domain and a cytoplasmic domain. M-PAPC: membrane bound PAPC mutant lacks most of the cytoplasmic domain, compared to FL-PAPC. PAPC-TMC: PAPC extracellular domain deletion mutant only contains a signal peptide, a short linker, and the PAPC transmembrane domain and cytoplasmic domain. FLRT3: the full-length FLRT3 has a signal peptide, an extracellular domain composed of a Leucine-Rich-Repeat (LRR) region and a fibronectin-III (FNIII) domain, a transmembrane domain and a cytoplasmic region capable of binding RND1; both Flag-tagged and non-tagged FLRT3 were used. FLRT3-TMC: the extracellular domain deletion mutant of FLRT3 contains a signal peptide, a short linker, and the FLRT3 transmembrane domain and cytoplasmic domain with a Flag-tag. RND1-HA: N-terminal HA-tagged RND1.
(7.73 MB TIF)

Figure S2 C-cadherin remains at the cell surface after FLRT3 expression. A) The effect of FLRT3 expression on the accessibility of C-cadherin to trypsin treatment of the cell surface. Blastomeres collected from stage 9 embryos that had been injected with either GFP RNA (as control) or FLRT3 RNA (0.2 ng) at the 4 cell stage were either mock-treated or treated with trypsin/EDTA. Samples were analyzed by western blotting against C-cadherin and FLRT3. Duplicates were independent experiments. B) The effect of activin treatment, FLRT3 expression, or PAPC expression on the accessibility of C-cadherin to cell surface biotinylation. Stage 9 blastomeres, which were either untreated (Con), treated with 5 ng/ml activin for 1 hr, or from embryos that had been injected with 0.2 ng of FLRT3 RNA (FLRT3) or 1.5 ng of FL-PAPC (PAPC) at 4-cell stage, were incubated with surface biotinylation reagent, NHS-sulfo-s-s-Biotin. The labeled surface proteins were pulled down with neutravidin beads and analyzed by western blotting against C-cadherin. Each lane of triplet is an independent experiment. C) The quantification of the percentage of biotinylated C-cadherin in samples from B. D) Anti-C-cadherin confocal immunofluorescence microscopy of the dorsal marginal zone cryosection of a stage 12 embryo. ec  =  ectoderm; me  =  mesoderm; en  =  endoderm; yp  =  yolk plug.
(9.14 MB TIF)

Figure S3 FLRT3 morpholinos effectively knocks down FLRT3 expression in Xenopus embryos. FLRT3 RNA (0.5 ng) was injected with or without 40 ng of FLRT3 morpholinos (FLRT-MO) into the embryos at the 2 cell stage. At stage 9, the embryos were lysed and the protein extracts were analyzed by SDS-PAGE and western blots against FLRT3. GFP RNA (0.5 ng) injected embryos were used as control to show that no endogenous FLRT3 was detectable by western blot at stage 9. Duplicate samples in the gel were from independent experiments.
(1.89 MB TIF)

Figure S4 FL-PAPC and M-PAPC, but not PAPC-TMC (transmembrane+cytoplasmic domain), can rescue FLRT3-TMC induced cell dissociation. 1 ng of control (GFP) RNA (A and E), FL-PAPC RNA (B and F), M-PAPC RNA (C and G) or PAPC-TMC RNA (D and H) was co-injected with 0.25 ng of FLRT3-TMC RNA into the animal hemispheres of 4-cell stage embryos. At stage 9, pictures of the outer (A–D) and inner (E–H) surfaces of the animal cap region or explants of the injected embryos were taken and the severity of the cell adhesion disruption was compared.
(8.64 MB TIF)

Figure S5 Detection of PAPC, FLRT3 and C-cadherin interactions by crosslinking and co-IP. Total membranes prepared from stage 10.5 embryos that were injected with PAPC and FLRT3 RNAs were crosslinked with 0.5 mM Sulfo-DST, dissolved in Ripa buffer, and IPed with preimmune IgG, anti-PAPC IgG, or anti-FLRT3 IgG and analyzed by Western blotting for PAPC, C-cadherin, FLRT3 or integrin α5 (as negative control).
(1.89 MB TIF)

Figure S6 Immunofluorescence staining of integrin α5 in A431 cells. Integrin a5 antibody effectively stained the focal adhesion foci and lamellipodia in A431 cells.
(7.45 MB TIF)
