# Supplementary figures and images for: A Protocadherin-Cadherin-FLRT3 Complex Controls Cell Adhesion and Morphogenesis
Source: PLoS One. 2009 Dec 22;4(12):e8411. doi: 10.1371/journal.pone.0008411 (PMC2791867; doi:10.1371/journal.pone.0008411)

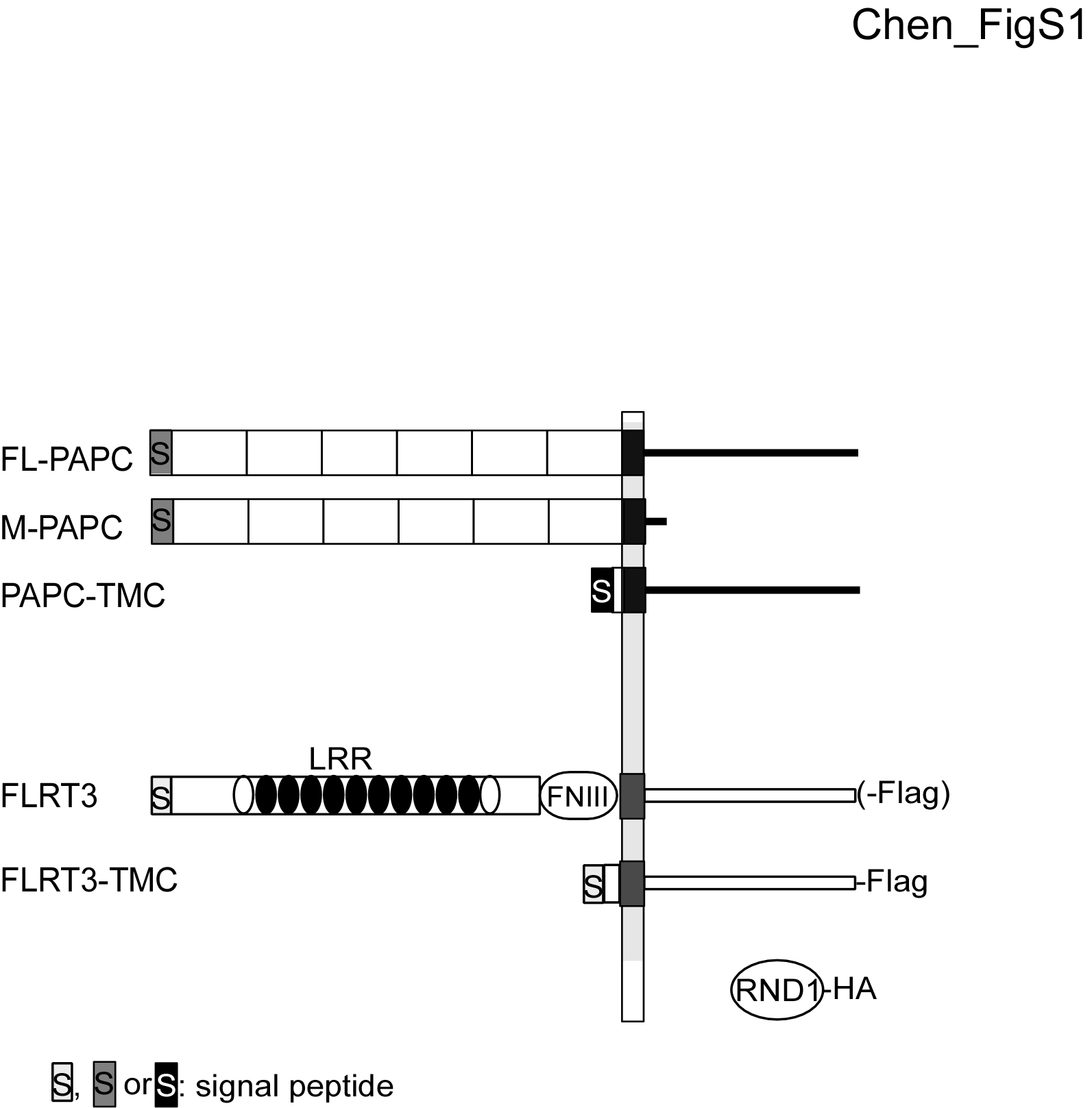

Supplement: Figure S1 — Scheme of different protein constructs used in this study. FL-PAPC: the full-length PAPC contains a signal peptide, 6 cadherin ectodomains, a transmembrane domain and a cytoplasmic domain. M-PAPC: membrane bound PAPC mutant lacks most of the cytoplasmic domain, compared to FL-PAPC. PAPC-TMC: PAPC extracellular domain deletion mutant only contains a signal peptide, a short linker, and the PAPC transmembrane domain and cytoplasmic domain. FLRT3: the full-length FLRT3 has a signal peptide, an extracellular domain composed of a Leucine-Rich-Repeat (LRR) region and a fibronectin-III (FNIII) domain, a transmembrane domain and a cytoplasmic region capable of binding RND1; both Flag-tagged and non-tagged FLRT3 were used. FLRT3-TMC: the extracellular domain deletion mutant of FLRT3 contains a signal peptide, a short linker, and the FLRT3 transmembrane domain and cytoplasmic domain with a Flag-tag. RND1-HA: N-terminal HA-tagged RND1. (7.73 MB TIF) [file pone.0008411.s001.tif]

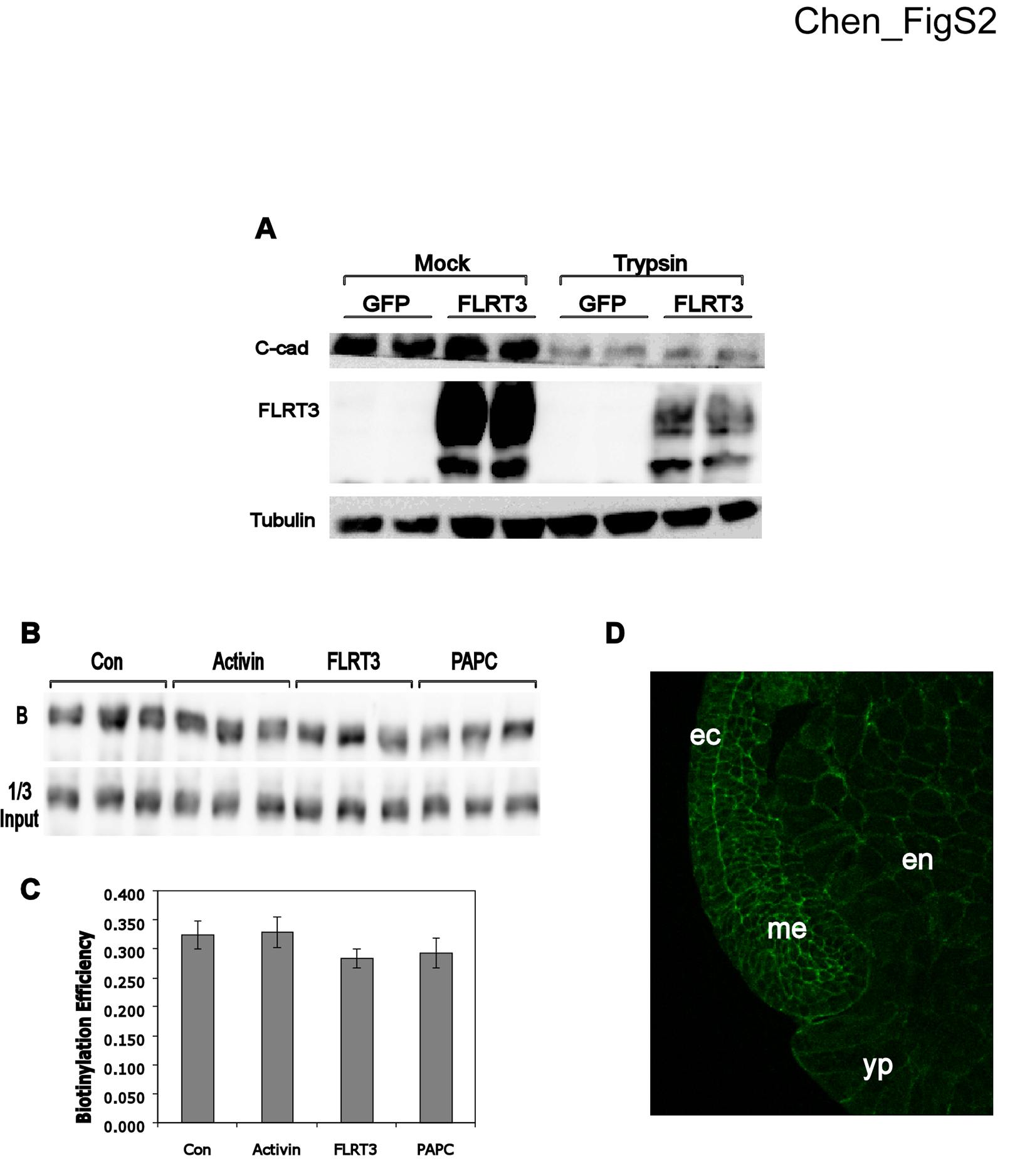

Supplement: Figure S2 — C-cadherin remains at the cell surface after FLRT3 expression. A) The effect of FLRT3 expression on the accessibility of C-cadherin to trypsin treatment of the cell surface. Blastomeres collected from stage 9 embryos that had been injected with either GFP RNA (as control) or FLRT3 RNA (0.2 ng) at the 4 cell stage were either mock-treated or treated with trypsin/EDTA. Samples were analyzed by western blotting against C-cadherin and FLRT3. Duplicates were independent experiments. B) The effect of activin treatment, FLRT3 expression, or PAPC expression on the accessibility of C-cadherin to cell surface biotinylation. Stage 9 blastomeres, which were either untreated (Con), treated with 5 ng/ml activin for 1 hr, or from embryos that had been injected with 0.2 ng of FLRT3 RNA (FLRT3) or 1.5 ng of FL-PAPC (PAPC) at 4-cell stage, were incubated with surface biotinylation reagent, NHS-sulfo-s-s-Biotin. The labeled surface proteins were pulled down with neutravidin beads and analyzed by western blotting against C-cadherin. Each lane of triplet is an independent experiment. C) The quantification of the percentage of biotinylated C-cadherin in samples from B. D) Anti-C-cadherin confocal immunofluorescence microscopy of the dorsal marginal zone cryosection of a stage 12 embryo. ec = ectoderm; me = mesoderm; en = endoderm; yp = yolk plug. (9.14 MB TIF) [file pone.0008411.s002.tif]

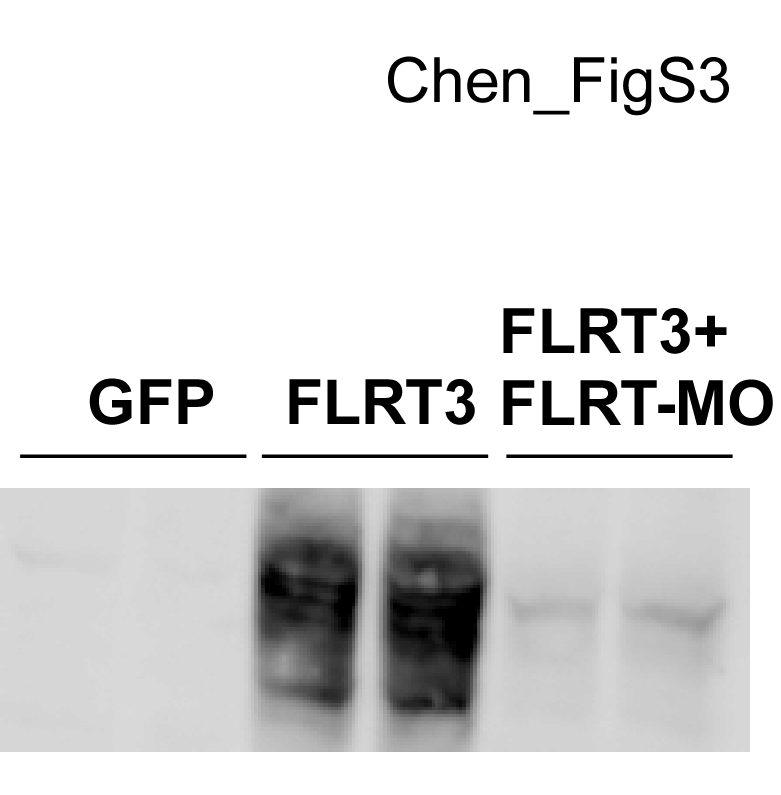

Supplement: Figure S3 — FLRT3 morpholinos effectively knocks down FLRT3 expression in Xenopus embryos. FLRT3 RNA (0.5 ng) was injected with or without 40 ng of FLRT3 morpholinos (FLRT-MO) into the embryos at the 2 cell stage. At stage 9, the embryos were lysed and the protein extracts were analyzed by SDS-PAGE and western blots against FLRT3. GFP RNA (0.5 ng) injected embryos were used as control to show that no endogenous FLRT3 was detectable by western blot at stage 9. Duplicate samples in the gel were from independent experiments. (1.89 MB TIF) [file pone.0008411.s003.tif]

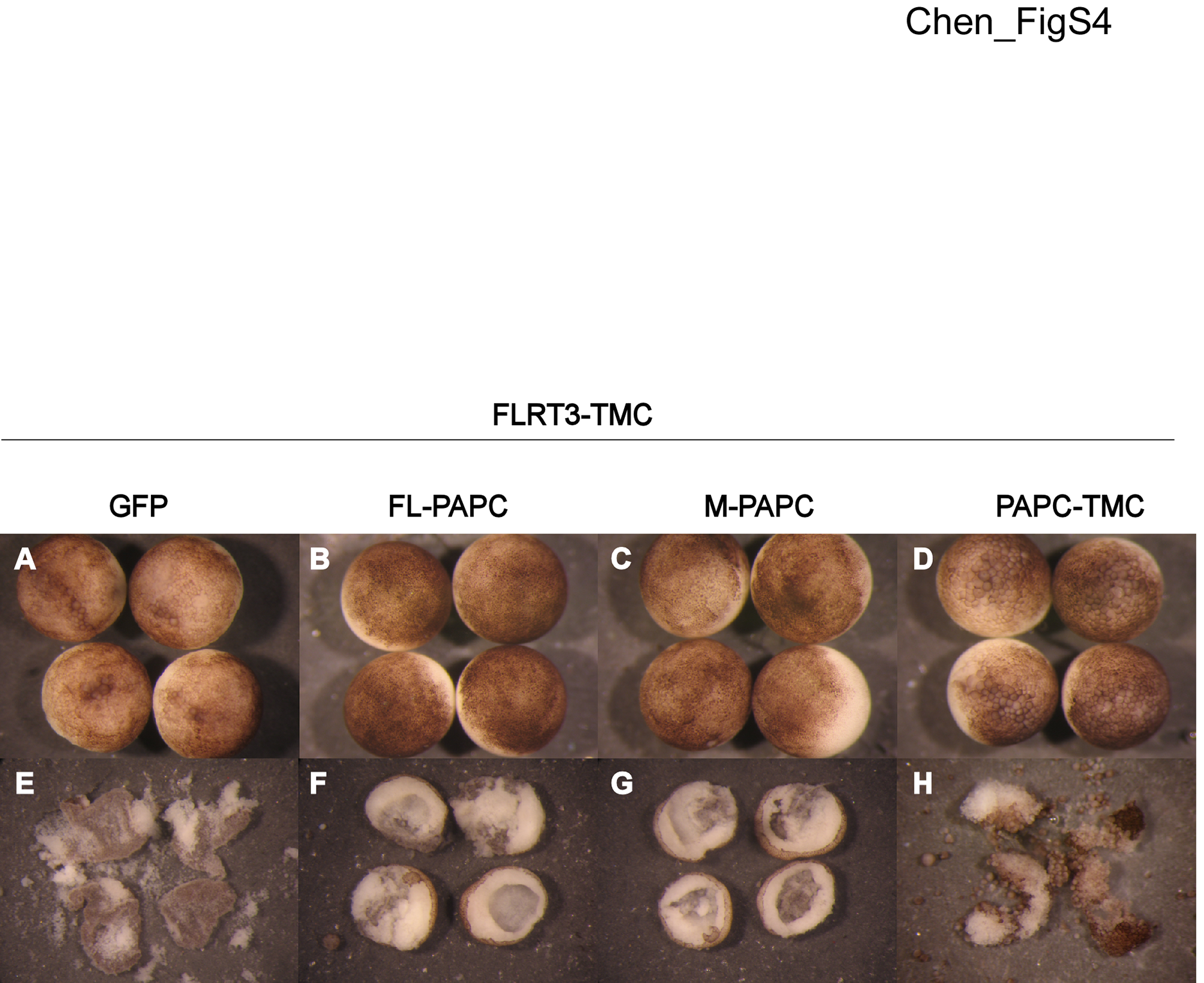

Supplement: Figure S4 — FL-PAPC and M-PAPC, but not PAPC-TMC (transmembrane+cytoplasmic domain), can rescue FLRT3-TMC induced cell dissociation. 1 ng of control (GFP) RNA (A and E), FL-PAPC RNA (B and F), M-PAPC RNA (C and G) or PAPC-TMC RNA (D and H) was co-injected with 0.25 ng of FLRT3-TMC RNA into the animal hemispheres of 4-cell stage embryos. At stage 9, pictures of the outer (A–D) and inner (E–H) surfaces of the animal cap region or explants of the injected embryos were taken and the severity of the cell adhesion disruption was compared. (8.64 MB TIF) [file pone.0008411.s004.tif]

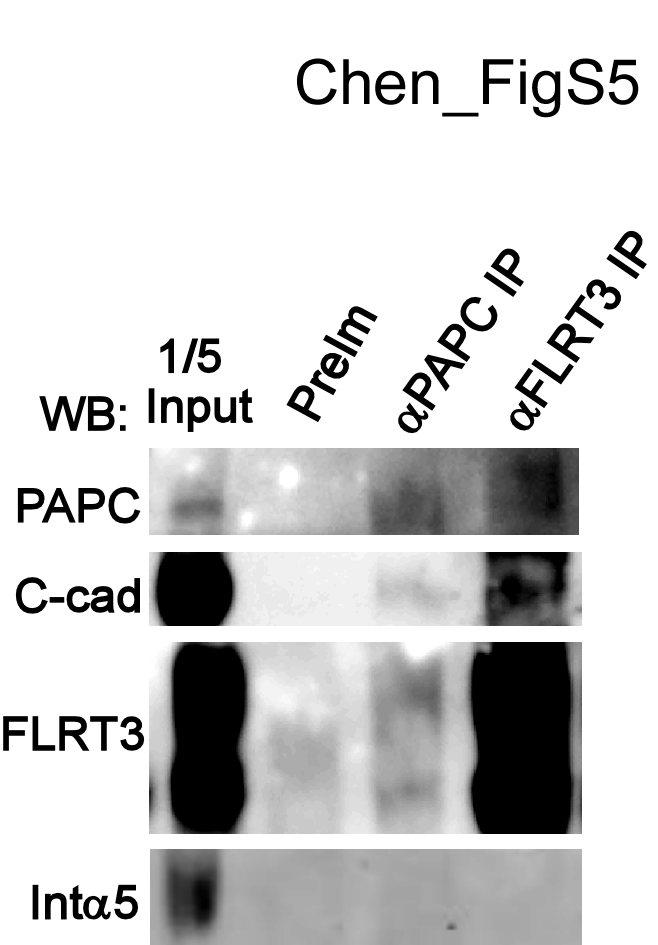

Supplement: Figure S5 — Detection of PAPC, FLRT3 and C-cadherin interactions by crosslinking and co-IP. Total membranes prepared from stage 10.5 embryos that were injected with PAPC and FLRT3 RNAs were crosslinked with 0.5 mM Sulfo-DST, dissolved in Ripa buffer, and IPed with preimmune IgG, anti-PAPC IgG, or anti-FLRT3 IgG and analyzed by Western blotting for PAPC, C-cadherin, FLRT3 or integrin α5 (as negative control). (1.89 MB TIF) [file pone.0008411.s005.tif]

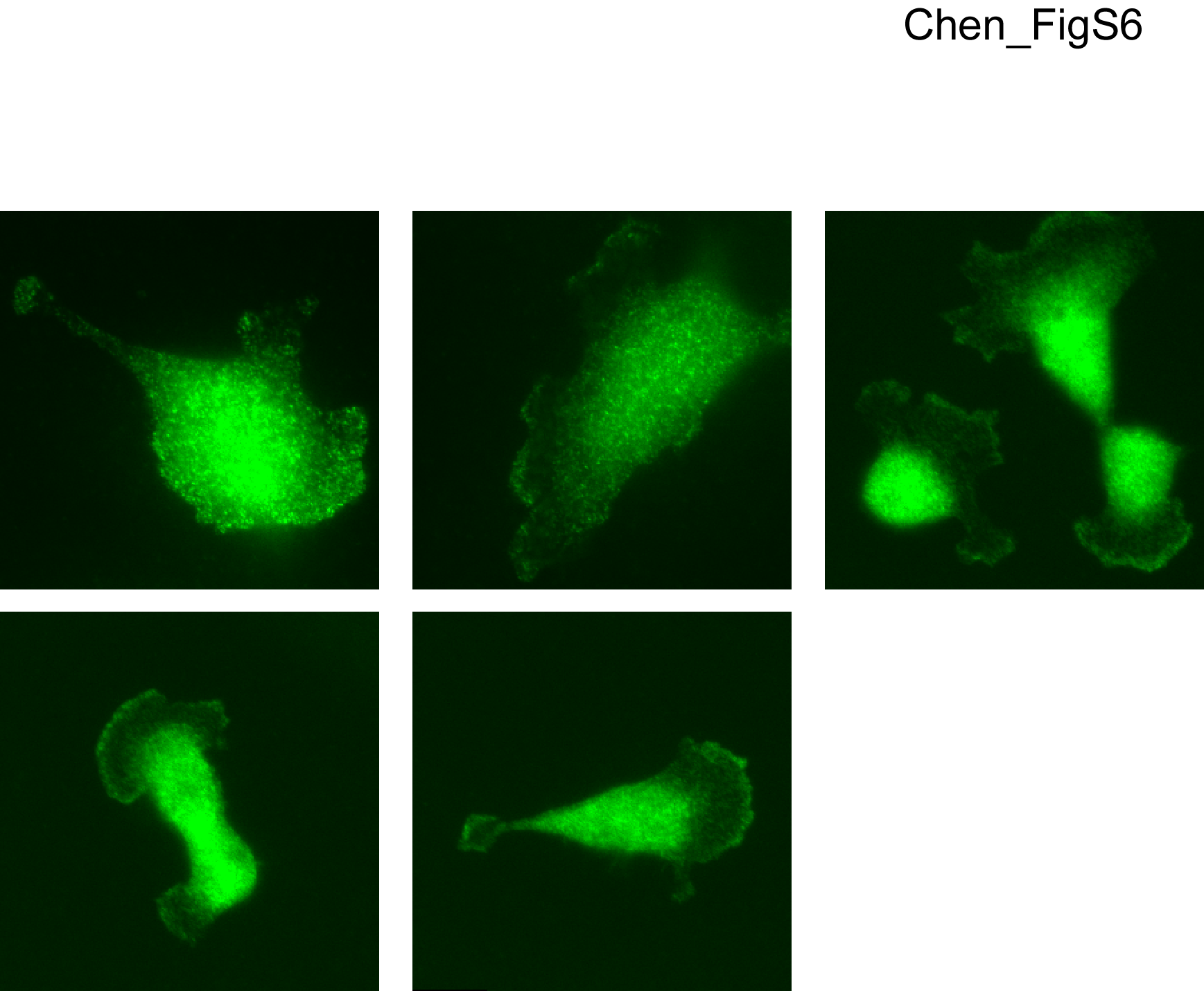

Supplement: Figure S6 — Immunofluorescence staining of integrin α5 in A431 cells. Integrin a5 antibody effectively stained the focal adhesion foci and lamellipodia in A431 cells. (7.45 MB TIF) [file pone.0008411.s006.tif]
